# Design Patterns for the Development and Implementation of Bioacoustic Deep Learning Recognizers

**DOI:** 10.1002/ece3.74030

**Published:** 2026-08-04

**Authors:** Gavin Hurd, Robin Baron, Jesse Whittington

**Affiliations:** ^1^ Parks Canada Agency, Government of Canada Banff Alberta Canada

## Abstract

Bioacoustic monitoring using autonomous recording units generates volumes of audio data that exceed the capacity of manual annotation, necessitating the use of automated species recognizers. Convolutional Neural Networks (CNNs) have emerged as the dominant approach due to their strong performance on spectrogram representations of audio data, yet their development and deployment remain challenging. This paper presents a set of reusable design patterns that address recurring methodological challenges in two key areas: (1) CNN recognizer development and (2) integration of recognizers into practical bioacoustics workflows. Drawing on a case study involving the development and implementation of a single‐species CNN recognizer for the western toad in Banff National Park, Canada, we illustrate patterns related to data leakage, sampling bias, signal processing decisions, hyperparameter optimization, model training, and workflow integration. Each pattern is described in a structured problem/solution format and supported with real‐world examples. Collectively, these patterns provide a comprehensive framework spanning recognizer design, deployment, and even iterative improvement through user interfaces and active learning. By formalizing best practices, this work aims to improve the reliability, efficiency, and accessibility of CNN‐based bioacoustic monitoring across a wide range of ecological applications.

## Graphical abstract

This paper presents a set of design patterns for developing and integrating Convolutional Neural Network (CNN) recognizers in bioacoustic monitoring programs. Using a western toad case study, we illustrate solutions to common challenges in model development and workflow integration. These patterns provide a practical framework to improve the efficiency, reliability, and accessibility of automated species recognition across ecological applications.

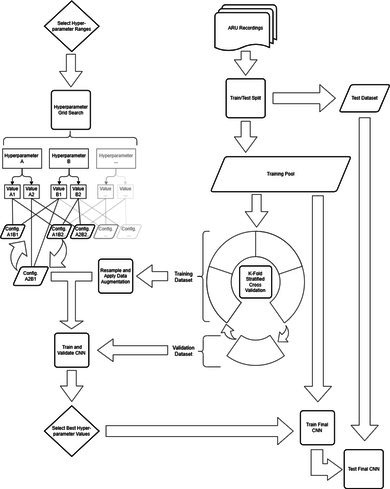

## 1 Introduction

Bioacoustic surveys are increasingly used to collect data for a diverse array of sound‐producing animal taxa and ecological applications (Brooker et al. 2020; Stowell 2022). The adoption of autonomous recording units (ARUs) has expanded rapidly due to their ability to create permanent survey records, reduce observer bias, reduce field effort, and improve scalability (Knight et al. 2020; Shonfield and Bayne 2017). However, the capacity to extend sampling area and duration with ARUs significantly surpasses the rate at which human observers can annotate audio recordings (MacPhail et al. 2024). In response, automated computer detection algorithms, referred to as “recognizers,” have been developed to identify the vocalizations of species (Knight et al. 2017). Among these, Convolutional Neural Networks (CNNs) have become the de facto standard due to their superior performance and their ability to integrate traditionally manual pre‐processing steps directly into an automated feature extraction process (Brown 2024; Stowell 2022).

Several platforms have been developed to facilitate the use of recognizers, such as BirdNET (Kahl et al. 2021), Arbimon (Aide et al. 2013), WildTrax (Alberta Biodiversity Monitoring Institute and Bioacoustic Unit 2024), and Raven Pro (K. Lisa Yang Center for Conservation Bioacoustics 2026, Cornell University; https://doi.org/10.1111/2041‐210x.70285). However, these platforms do not support all species or ecological applications (Brooker et al. 2020; Brown 2024). Furthermore, recognizers trained on site‐specific recordings may outperform models derived primarily from global or public repositories because they better capture local acoustic conditions, habitat‐specific attenuation, specific vocalization types, regional dialects, and confounding environmental sounds unique to a monitoring location (Huus et al. 2024). Consequently, bioacoustic practitioners may benefit from adapting pre‐built models through transfer learning or by developing custom‐built models for their specific species and objectives (Brown 2024).

This paper is intended as a practical bridge between ecology and applied machine learning for bioacoustic practitioners who may increasingly develop, adapt, or deploy CNN recognizers without formal AI training. The development of CNN recognizers draws upon concepts from machine learning, audio processing, computer engineering, and mathematics that may be unfamiliar to practitioners trained primarily in ecology (Brooker et al. 2020). As advances in software, pretrained models, and AI‐assisted coding continue to lower technical barriers, the risk of poorly designed or insufficiently validated workflows may also increase. This study presents a series of design patterns that address recurring challenges and common methodological oversights encountered during recognizer development and implementation.

**FIGURE 1:**
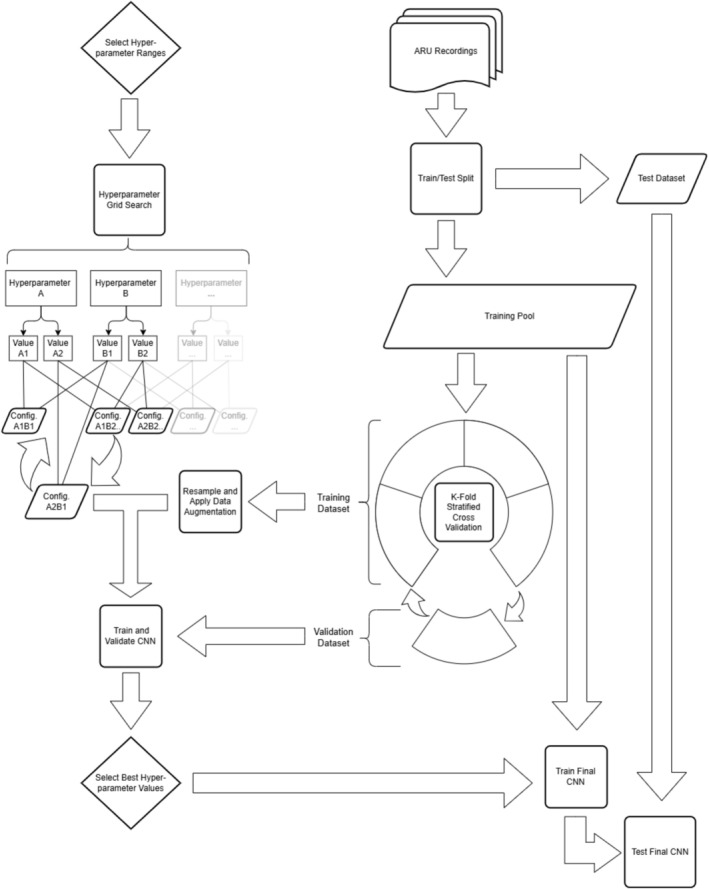
Overview of the CNN recognizer development workflow for western toad classification, including dataset partitioning, K‐fold cross‐validation for hyperparameter tuning and model selection, final model training, and evaluation using a withheld test dataset.

Design patterns have been used to address recurring challenges in methodological processes (Greenberg et al. 2019). In bioacoustics, challenges often emerge in two disparate areas: (1) the development of CNN recognizers, and (2) the integration of recognizers into bioacoustic workflows (Shonfield and Bayne 2017; Stowell 2022). The design patterns presented in this study provide structured templates and reusable solutions to commonly occurring problems within these areas. Each pattern will be illustrated with a real‐world example drawn from the development and implementation of a custom‐built western toad ( *Anaxyrus boreas* ) CNN recognizer in Banff National Park, Canada. Collectively, these patterns form a framework that captures the full development and implementation of a CNN‐based bioacoustic recognizer (Figure 1). These same design patterns can also be applied within transfer learning workflows, where pretrained CNNs are adapted to new species, regions, or acoustic environments using comparatively small labeled datasets (Ghani et al. 2023; White et al. 2023). This can reduce annotation effort while still benefiting from site‐specific optimization. The design patterns may also support active learning workflows, in which iterative model‐assisted annotation is used to efficiently expand training datasets, address confounding sounds, and improve recognizer performance over time (Kath et al. 2024; Márquez‐Rodríguez et al. 2026).

## 2 General Methodology

Our methodology for identifying useful design patterns was informed through a western toad monitoring project in Banff National Park. The aim of this project was to estimate long‐term changes in the spatial distribution of western toads. We developed a single‐species CNN recognizer and streamlined workflows through a user interface. Many challenges incurred during this project are likely to arise in other bioacoustic projects, such as balancing automation with analyst oversight. The design patterns presented here aim to provide generalizable solutions intended for a broad range of applications including multi‐species recognizers.

The generalizability of design patterns stems from their focus on identifying and addressing underlying forces within a design problem (Marinescu 2002). We present each design problem in Alexandrian form, starting with a description of the context and nature of the problem followed by the proposed solution (Alexander et al. 1977; Marinescu 2002). For each design pattern, we included western toad examples to illustrate a practical application. This structure is intended to equip bioacoustic practitioners with the knowledge and insight needed to adapt these solutions to future bioacoustics challenges.

## 3 CNN Recognizer Development

**FIGURE 2:**
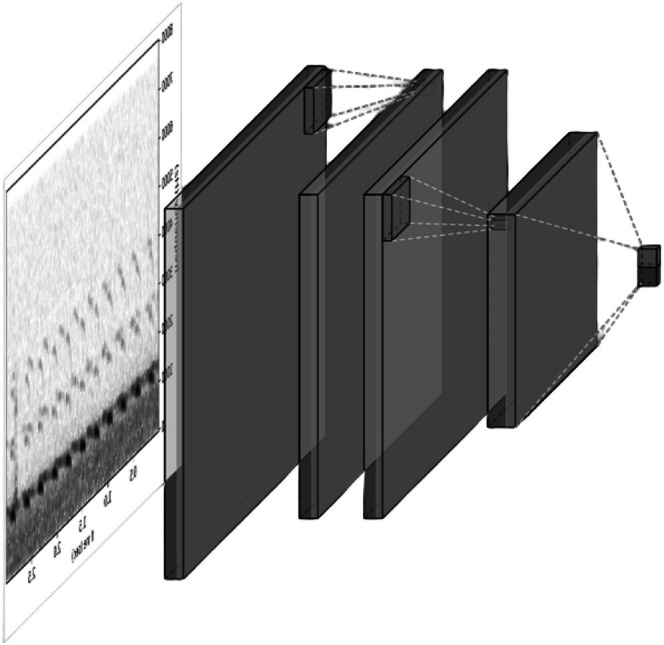
Conceptual diagram of a convolutional neural network for spectrogram‐based bioacoustic classification. The model takes a spectrogram of an acoustic signal as input and processes it through successive convolutional feature‐extraction stages, where learned filters detect local patterns such as edges, pulses, and frequency structure. These features are combined across deeper layers to form increasingly abstract representations, enabling hierarchical feature learning from low‐level acoustic cues to species‐relevant patterns.

CNN recognizers are a class of artificial neural networks designed to identify patterns within grid‐structured data such as images (Goodfellow et al. 2016). In bioacoustics, audio recordings can be converted into spectrograms, which visually represent the frequency and amplitude structure of sound over time (Stowell 2022). CNNs analyze these spectrograms by applying a series of filters that detect localized visual features, pulses, or call shapes, and progressively combine them into higher‐level acoustic patterns useful for classification (Figure 2; Goodfellow et al. 2016; Zhao et al. 2024). This hierarchical feature‐learning process allows CNNs to automatically extract informative acoustic characteristics directly from spectrograms without requiring manual feature engineering, such as the calculation of Mel‐frequency cepstral coefficients (Brown 2024). As a result, CNN recognizers can operate in much higher dimensional spaces, enabling them to distinguish between subtle variations in similar acoustic signals (Stowell 2022).

The use of high dimensional data in the development of CNN recognizers requires considerable computational power and time. For example, Brown (2024) noted that a single CNN recognizer training iteration can take 3.5 h on a powerful graphics processing unit. However, this up front cost is mitigated by subsequent rapid acoustic recording processing of new data, particularly in comparison to recognizer models that rely on costly pre‐processing, segmentation, and acoustic feature extraction (Brown 2024).

Given the resources required to develop a CNN recognizer, minimizing methodological errors and streamlining model development help prevent the unnecessary repetition of steps in the development process. While the conceptual framework for developing a CNN recognizer is straightforward—train a CNN to identify a species, tune the hyperparameters, and evaluate the final model—the process can often be complicated by challenges such as limited data and limited computational resources, acoustic signal processing complexities, and a lack of familiarity with machine learning best practices. The design patterns discussed in the following sections are intended to provide guidance and structure to the development of a CNN recognizer, facilitating the enactment of standardized and reliable practices.

### 3.1 Data Leakage

Data leakage occurs when information from the testing set—the withheld data used to evaluate the final model—unintentionally influences the development of the model, creating an illusion of high performance that fails in practice. This issue is so prevalent it has led to the declaration of a reproducibility crisis in machine‐learning based science (Kapoor and Narayanan 2022). A common cause is the use of overlapping data; Stowell (2022) notes that many bioacoustics studies use the same recordings for training and testing. In these cases, a model may show near‐perfect accuracy because it has “memorized” specific files rather than learning general species characteristics.

A more subtle form of data leakage occurs when parameters, such as the window size for a Short‐Time Fourier Transform or confidence thresholds, are selected using the full dataset or optimized specifically for the testing set. In the latter case, selecting a threshold that maximizes performance on the test data incorporates information that should remain unseen during development. This introduces a hidden bias that inflates performance metrics; however, this advantage collapses in new environments and can result in invalid conclusions (Kapoor and Narayanan 2022; Starcke et al. 2025).

#### 3.1.1 Design Pattern

The partitioning of labeled data into training, validation, and testing partitions is best informed by their fundamental purposes: training data is used to teach the CNN to identify patterns in acoustic signals. The primary focus of a training dataset is to provide many classified samples of the vocalizations and the background acoustic environment; this provides an opportunity for the CNN to learn how to recognize patterns and variations within the acoustic signals and to differentiate signals from the acoustic environment. The validation dataset then helps tune the hyperparameters by comparing predictions from different training regimes to the classified samples in the validation dataset. Because the validation dataset is used repeatedly to compare different hyperparameter configurations, a form of data leakage is introduced. To address this, K‐fold cross‐validation—where the data are repeatedly partitioned into different training and validation subsets across multiple iterations—can improve the reliability of hyperparameter selection and reduce the risk of overfitting (Stowell et al. 2019). Similarly, the testing dataset is necessary to provide an unbiased estimate of the CNN recognizer's real‐world performance and account for the possibility that the CNN has been overfit to the validation dataset.

#### 3.1.2 Example

We developed a western toad CNN recognizer using audio recordings collected between 2021 and 2023 with automated recording units (ARUs, Song Meter Mini; Wildlife Acoustics Inc. Concord, MA, USA) deployed at over 20 ponds in Banff National Park. We deployed the ARUs during May and June during the breeding season and programmed them to record vocalizations for 3 min every hour, between 8 p.m. and 4 a.m. After retrieval, we manually transcribed audio. Transcription involved listening for western toad calls and, when present, noting their occurrence at multiple instances per recording. ARUs also recorded abiotic sounds such as heavy wind, vehicles, and trains within the background environment. We split audio recordings into three‐second segments, referred to as “clips”. We labeled clips with western toad vocalizations as positive and all other clips as negative.

We first randomly partitioned audio clip recordings into training and validation datasets (training pool; 90%) and testing (10%) data. Next, we randomly grouped audio recordings from the training pool to create five validation folds that contained approximately 20% of the total clips in the training pool. This approach mitigated the risk of data leakage from confounding variables. Following these partitions, the training pool consisted of 644 positives and 1332 negative clips, while the testing dataset included 81 positive and 84 negative clips.

### 3.2 Clip Ratio Bias in Recognizer Development and Evaluation

Model development and evaluation are both influenced by how frequently each species appears in the dataset (hereafter “clip ratio”; Kapoor and Narayanan 2022). In a binary recognizer—a model that classifies inputs into one of two categories (e.g., single‐species presence or absence)—this clip ratio refers to the proportion of positive and negative classes. Under imbalanced clip ratios (i.e., an equal representation of species in the training set), the optimization process is dominated by the majority‐species; the model reduces overall error by prioritizing these frequent patterns, often at the expense of learning the rare features of minority species. In extreme cases, models may converge to degenerate solutions that predict the most common species by default (Johnson and Khoshgoftaar 2019; Leevy et al. 2018). As a result, the model may converge on a solution that fails to accurately detect less common species. This issue has been widely observed and persists regardless of dataset scale, reflecting the intrinsic difficulty of learning under imbalanced clip ratios.

If clip ratio imbalances persist in the validation dataset, hyperparameter optimization may favor models that perform well on the most common species, leading to suboptimal configurations that systematically underperform on less frequent species (Leevy et al. 2018). If the testing dataset also has a clip ratio imbalance, reported performance metrics may appear strong while potentially obscuring substantial variability in per‐species accuracy (Kapoor and Narayanan 2022). Consequently, these aggregate metrics may fail to predict performance in real‐world operational environments, where species vocalization ratios often differ significantly from those in the evaluation datasets.

The issue is particularly relevant for multi‐species recognition models, where performance can vary substantially across species (Singer et al. 2024). In such cases, aggregate metrics can mask large disparities in per‐species performance, giving an incomplete or overly optimistic assessment of CNN recognizer capability (Schneider et al. 2020; Zeng 2025). In contrast, for binary CNN recognizers, imbalance manifests differently but remains consequential. When negative clips dominate, metrics such as accuracy and specificity may appear strong even if recall on the rarer positive class is poor. This can result in models that systematically miss rare but important events (Thölke et al. 2023).

#### 3.2.1 Design Pattern

During training, bioacoustic practitioners can control the effects of clip ratios by resampling or differentially weighting clips (Stowell 2022). Resampling adjusts the proportion of clips to achieve more balanced clip ratios, by either removing the instances of the majority class or duplicating minority class clips (Thölke et al. 2023). When resampling with replacement to duplicate clips, bioacoustic data augmentation techniques such as time shifting and frequency masking can be employed to alleviate the risk of overfitting (Stowell 2022). Alternatively, the effects of unequal clip ratios in the training dataset can be controlled by per‐species weighting within the training algorithm optimizer (e.g., the loss function), to create an imbalance‐aware loss function (Zbinden et al. 2024). Furthermore, per‐species weighting allows the CNN training process to be tailored to specific project goals. For example, it can be used to increase the detection sensitivity for rare species in a multi‐species model, or to prioritize higher precision by placing greater weight on negative clips (false positives) in a binary recognizer (Dong et al. 2021).

Clip ratios must be carefully considered when evaluating CNN recognizer performance (both during validation and testing). Any resampling of the validation or testing datasets should be performed without replacement to avoid duplication bias, where repeated inclusion of the same clips can distort performance estimates by reducing the independence of evaluation samples. For a generalizable estimate of a CNN recognizers' performance, the testing dataset should reflect the real‐world species distribution of the study area (Kapoor and Narayanan 2022). For example, a species that is rarely encountered should have a proportionally small influence on performance metrics unless it is of specific interest to the researchers. However, because real‐world distributions of species within a soundscape are often unknown, aggregate evaluation metrics must be interpreted with caution, and it is often advisable to ensure all species are aggregated equally (Chen et al. 2025).

One strategy to mitigate clip ratio effects is macro‐averaging, which calculates metrics independently for each class before averaging the results, thereby giving equal weight to rare and common species (Mesaros et al. 2016). This framework is important for standard metrics like precision, recall, and the F1‐score, as it prevents the aggregate performance from being dominated by the most prevalent classes (Aguilar‐Ruiz and Michalak 2024). While the Area Under the Receiver Operating Characteristic curve (AUC‐ROC) is insensitive to clip ratios in binary classifications, its extension to multispecies classifiers is more complex (Xu 2024). In the multispecies context, AUC‐ROC is no longer a single calculation but rather a collection of scores that requires an aggregation strategy such as macro‐averaging to maintain insensitivity to imbalanced clip ratios (Hand and Till 2001). Standard One‐vs.‐Rest macro‐averaging can still exhibit bias in imbalanced datasets because it pools all non‐target species into a single class dominated by the most common species (Giudici et al. 2025). In contrast, One‐vs.‐One macro‐averaging decomposes the evaluation into every possible pairwise combination, which can help ensure that per‐species metrics are equally represented in the final score (Hand and Till 2001).

#### 3.2.2 Example

We employed five‐fold stratified cross‐validation at the recording level to account for within‐recording correlations. This process involves partitioning the data into five subsets (folds), sequentially withholding one for validation while training the model on the remaining four. To mitigate the effects of uneven clip ratios in our training pool (644 positive; 1332 negative), we used resampling with replacement to generate a balanced training dataset of 1000 clips per class. The training dataset sample size represented a practical compromise between maximizing acoustic diversity and limiting excessive duplication of minority clips during resampling. To alleviate the risk of overfitting during this resampling, we applied standard bioacoustic data augmentation, including time shifting and frequency masking. Alternative balanced training dataset sizes could be evaluated during hyperparameter optimization, or imbalance could be addressed through loss‐function reweighting using the original clip ratios. To prioritize precision and reduce false positives, we implemented a project‐specific loss function by scaling the weight applied to positive clips by 0.8. This hybrid approach aimed to ensure the CNN remained sensitive to the minority class while maintaining a high threshold for detections. We evaluated the model using the AUC‐ROC; this metric was selected for its insensitivity to clip ratios and decision thresholds in a simple binary context.

### 3.3 Calculation of a STFT Window Length

CNNs are optimized to analyze visual data; it is therefore necessary to convert acoustic clips into spectrogram images. This conversion is generally accomplished through a discrete short‐time Fourier transform (STFT; Stowell 2022; Knight et al. 2020). The main parameters in this transform are the length, type, and overlap of the STFT window. Spectrogram preprocessing may also include selecting a restricted frequency range to exclude irrelevant acoustic content and focus the representation on frequencies relevant to the target species.

**FIGURE 3:**
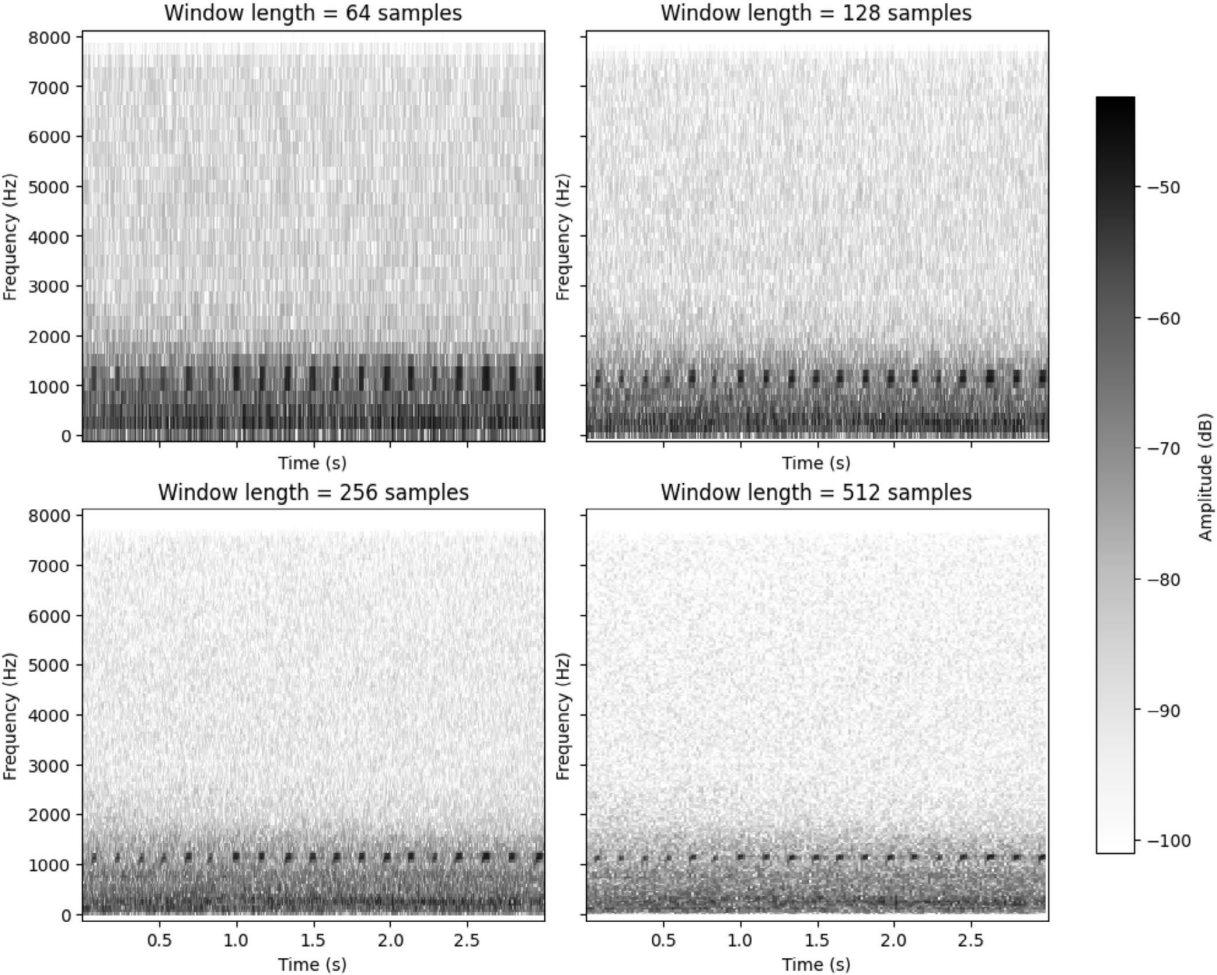
Spectrograms of a western toad vocalization generated using short‐time Fourier transforms (STFTs) with varying Hanning window lengths (64, 128, 256, and 512 samples) and 50% overlap at a 16 kHz sampling rate. Spectrograms were converted to decibel (dB) scale. Pixel intensity represents relative signal energy. Across window sizes, the same vocalization is represented with differing time–frequency resolution, with shorter windows emphasizing temporal precision and longer windows enhancing frequency resolution of harmonic structure. Western toad calls appear as short, repeated pulses with a dominant frequency centered at approximately 1100 Hz.

A Hanning window with 50% overlap is commonly used in spectrogram generation to ensure full coverage of the audio recording, and to reduce spectral leakage, which occurs when energy from one frequency spreads into adjacent frequency bins (Cerna and Harvey, n.d.; Trethewey 2000). However, the optimal window length depends on the characteristics of the acoustic signals, which in our case were amphibian calls (Knight et al. 2020). Longer windows provide better frequency resolution but reduce temporal resolution (Figure 3), forcing bioacoustic practitioners to find a balance appropriate for their species of interest (Stowell 2022). Additionally, window lengths that are powers of two are often preferred because they enable the use of fast Fourier transform algorithms (FFT), which improve computational efficiency (Knight et al. 2020).

Optimal window length is often selected through hyperparameter optimization, testing a range of values during model training and validation. However, the many possible parameter values can quickly overwhelm the available computation resources. Practitioners are therefore advised to propose initial window lengths based on characteristics of the species, such as typical call duration and the need to resolve fine‐scale frequency features.

#### 3.3.1 Design Pattern

Calculating an appropriate window length in advance can significantly reduce the scope of hyperparameter optimization. If either the time or frequency dimension of the spectrogram is more important for species detection, the problem can be approached as a constrained optimization. The first step is to prioritize the dimension with greater discriminative power for the species. Then, a minimum acceptable resolution in the secondary dimension can be estimated, which acts as an inequality constraint. An additional equality constraint, requiring that the window length is a power of two, ensures compatibility with FFT algorithms. The optimal window length can then be selected as the highest (frequency‐priority) or lowest (time‐priority) value that satisfies both constraints.

If known, care should be taken to ensure that a minimum acceptable resolution in the priority dimension is achieved; if it is not, the inequality constraint on the secondary dimension must be re‐formatted as a soft constraint with a graduating penalty. Furthermore, if spectrograms are resized to meet CNN input requirements or computational constraints, practitioners should additionally ensure that the resized representation still preserves the minimum acceptable resolution. Prior to spectrogram generation, additional preprocessing steps such as frequency‐range restriction may further improve acoustic signal representation by excluding irrelevant noise.

In transfer learning workflows, the window length may instead be constrained by the pretrained model input configuration. Similarly, for multi‐species recognizers, a generalized window length may be required to accommodate species with differing acoustic characteristics, although alternative approaches such as multi‐channel inputs using different window lengths may also be effective.

#### 3.3.2 Example

We used a STFT to create spectrogram images from audio clips. Prior to spectrogram generation, we applied a bandpass filter—a type of frequency‐range restriction—between 900 and 1400 Hz to restrict the analyzed frequency range to frequencies relevant to western toad vocalizations and reduce irrelevant acoustic content. We specified all STFT parameters directly without including any in the hyperparameter optimization. We implemented the generally acceptable combination of a Hanning window with 50% overlap (Trethewey 2000).

To calculate an optimal window length, we first prioritized the frequency dimension. This decision was based on the presence of other amphibian species in the study area that produced similar temporal call patterns, leading us to hypothesize that differences in frequency structure would provide the most reliable basis for species differentiation. To determine a minimum acceptable resolution in the time dimension (i.e., maximum acceptable window size), we estimated the shortest duration of a pulse from a western toad vocalization. Through visual observations of spectrograms, we found this to be approximately 0.02 s (Figure 3). Given the audio sampling rate of 16 kHz, this duration corresponded to 320 audio samples (0.02 s × 16,000 samples/s = 320 samples). We rounded this value down to the nearest power of two, 256 samples (where 2^8^ = 256), to establish the FFT window length parameter that did not exceed the maximum acceptable window size of the time dimension (320 samples), while maximizing the frequency resolution.

The resulting spectrograms were resized to 224 × 224 pixels (equivalent to samples) to meet CNN input requirements. Because each spectrogram represented a 3 s audio clip, this resizing produced an effective temporal resolution of approximately 0.013 s per pixel (3 s/224 pixels), which remained below the estimated minimum pulse duration of 0.02 s. We therefore considered the resized spectrograms to preserve sufficient temporal resolution to represent the relevant vocalization structure.

### 3.4 Hyperparameter Optimization Resource Requirements

Hyperparameter optimization (HPO) is a key component of machine learning and can significantly improve the performance of deep learning models by selecting the best model configuration from a range of hyperparameter spaces (Hutter et al. 2019). Hyperparameters are user‐specified model settings that control aspects of model structure or the learning process, such as learning rate, batch size, or STFT window length. Optimizing all hyperparameter values from first principles is rarely possible due to the complexity of the configuration space, a lack of information regarding the influence of the hyperparameters on the model, as well as conditionality between the hyperparameters (Hutter et al. 2019; Stowell 2022). As a result, automated HPO algorithms are commonly used, such as Bayesian optimization, surrogate models, and genetic algorithms (Hutter et al. 2019). However, these HPO algorithms can be computationally expensive.

#### 3.4.1 Design Pattern

Given the high cost of HPO, it is important to minimize unnecessary computation within each training instance. The duration of a CNN training instance can be measured in epochs, defined as a complete pass of the training data through the CNN. While additional epochs may improve performance in early training, continued training beyond the point of convergence can lead to overfitting and unnecessary computational expense. This creates a practical need for mechanisms that identify when further training is no longer beneficial.

Early stopping addresses this by treating training as a monitored process rather than a fixed‐duration procedure (Goodfellow et al. 2016). Model performance is evaluated on a held‐out validation dataset after each epoch, and progress is assessed through changes in a chosen performance metric. Training is terminated when improvement falls below a defined threshold over a fixed patience window, indicating that the model has likely reached a performance plateau. Early stopping therefore functions as both a computational efficiency mechanism and a safeguard against overfitting during repeated HPO evaluations.

#### 3.4.2 Example

We conducted a grid search across four hyperparameters. The default configuration consisted of a Residual Network (ResNet; He et al. 2015) architecture trained using the AdamW optimizer (Loshchilov and Hutter 2019). AdamW combines adaptive gradient estimation with decoupled weight decay regularization, improving generalization performance while reducing the need for a learning rate scheduler. We evaluated two ResNet variants to assess the trade‐off between model complexity and overfitting, as deeper architectures can capture more complex acoustic structure but may generalize poorly on smaller datasets. Three learning rates were additionally evaluated, as learning rate selection strongly influences optimization stability and convergence speed.

No‐regret data augmentations (i.e., augmentations unlikely to alter the semantic content of bioacoustic signals) were randomly applied to the training data by default, including spectrogram trimming and temporal shifting (Stowell 2022). Data augmentation is commonly used in bioacoustics to improve model robustness and reduce overfitting by exposing models to realistic variation in signal structure and recording conditions. Additional experimental augmentations included additive noise overlays and spectrogram rescaling. Similar approaches have been shown to improve robustness and classification performance in bioacoustic classifiers (Kumar et al. 2024).

The hyperparameter grid therefore consisted of combinations of augmentation strategy, learning rate, and ResNet variant, yielding 24 unique configurations (Table 1). For each configuration, we implemented five‐fold cross‐validation. Training was terminated either when early stopping criteria were met or after 100 epochs, whichever occurred first. Model performance was evaluated using sigmoid‐transformed binary cross‐entropy loss. Early stopping criteria were defined as no improvement in validation loss across 16 consecutive epochs.

**TABLE 1:** Hyperparameters and values included in HPO.

Hyperparameter	Values
Data augmentation: Rescale spectrogram	True; False
Data augmentation: Add noise to spectrogram	True; False
ResNet layers	34; 50
Learning rate	1e‐3; 1e‐4; 1e‐5

### 3.5 Training the Final Model

After identifying the optimal hyperparameters through cross‐validated grid search with early stopping, additional challenges can arise when training the final model. In line with neural scaling laws, CNN performance is expected to improve with increased training data (Hestness et al. 2017). It is therefore advantageous to train the final model with the entire training pool, combining both training and validation clips used during cross‐validation. However, each fold in the HPO process may have stopped training after a different number of epochs due to early stopping, making it unclear how many epochs to use for the final model. Moreover, applying early stopping would require withholding a subset of labeled data for validation, which contradicts the goal of maximizing training data. As a result, selecting an appropriate training duration for the final model requires an alternative strategy.

#### 3.5.1 Design Pattern

Regularization helps establish a training regime in which model performance plateaus rather than deteriorates. When no validation dataset is available, performance must be derived solely from the training dataset, which often leads to inflated estimates due to overfitting. Various techniques can mitigate overfitting. These include penalizing large parameter values (e.g., L1 and L2 regularization) and introducing random variation (e.g., data augmentation and dropout layers). The optimal regularization strength can be identified through cross‐validation, where the optimal value is identified as the lowest magnitude that prevents overfitting without impeding learning.

#### 3.5.2 Example

Following an initial round of model development, we conducted an active learning stage prior to final hyperparameter selection, following the general approach of Huus et al. (2025). This iterative process applied the same design patterns described above and improved representation of difficult cases and confounding noise conditions in the training data.

After completing the final HPO of the last active learning iteration, we selected the configuration with the lowest average validation loss. These hyperparameter values were then used as the default in a subsequent hyperparameter grid search focused on regularization. In this second search, we inserted a dropout layer before the final fully connected layer in the ResNet and tested four dropout percentages as a new hyperparameter.

For each dropout percentage, we performed five‐fold stratified grouped cross‐validation, training each fold for 80 epochs. To assess the stability of the training regime and detect overfitting, we fit a linear regression to the validation loss from epochs 20–80, excluding earlier epochs when the CNN was likely still in the rapid learning phase. We then averaged the slopes and y‐intercepts across folds for each dropout percentage. A higher slope was interpreted as an indicator of overfitting. We applied a threshold of 0.1 to the slope and selected the lowest dropout percentage that met this criterion. We then used this dropout percentage to train the final CNN model on the full training pool.

The model was trained for 60 epochs and evaluated on a withheld testing dataset, achieving an AUC‐ROC of 0.93, with precision of 0.91 and recall of 0.64 at the default threshold of 0.5. This reflects the positive class weighting scheme designed to prioritize precision and reduce false positives. Threshold optimization was not performed; instead, the workflow relies on confidence‐ranked outputs and a dynamic dual‐thresholding approach to support both manual and automated classification within the user interface, as outlined in the integration design patterns in the following section.

## 4 Integration of Recognizer Into Workflows

Many bioacoustic recognizers are implemented as a series of Python or R scripts (Stowell 2022). While these scripts support reproducibility, script‐only implementations may limit the accessibility and utility of the CNN recognizers for a broader userbase (Stowell 2022). The output of a CNN recognizer typically consists of scores that represent classifier confidence of species presence within sequential segments of a recording (hereafter “predictions”). Regardless of the accuracy or reliability of a CNN recognizer, many bioacoustic workflows primarily use these predictions to subset and extract candidate audio clips for manual review (Pérez‐Granados 2023; Vernasco et al. 2025). The need for analysts to programmatically identify, extract, and review clips, sometimes even generating audio playback and spectrograms, can reduce the efficiency and consistency of bioacoustic workflows.

User interfaces (UIs) have been recognized as a key component for effectively integrating recognizers into bioacoustic workflows (Stowell 2022). A well‐designed UI can serve as an intermediary between the analyst and the recognizer, facilitating interaction and improving accessibility for non‐technical users. At a minimum, a bioacoustic UI must host the recognizer and allow users to generate predictions from selected audio recordings.

Beyond the basic functional requirements for bioacoustic UIs, there is no clear consensus on what constitutes the optimal UI design for bioacoustic applications (Stowell 2022). The complexity of working with large hierarchical datasets, integrating multi‐modal displays, and supporting flexible workflows presents unique design challenges. The following section explores UI features that can improve both the efficiency and accuracy of bioacoustic workflows. To inform this discussion, we developed design patterns during the creation of a UI for the western toad project. These patterns are intended to be flexible and can be adapted for other bioacoustic projects. By sharing them, we aim to offer practical insights for developing customizable, user‐friendly interfaces across a wide range of bioacoustic projects.

### 4.1 Navigating Hierarchical Datasets

Bioacoustic projects often involve large datasets organized across multiple hierarchical scales. CNN recognizers typically process these datasets by segmenting full audio recordings, creating at least two levels: complete recordings and their constituent clips. Some projects further group recordings by spatial and temporal attributes, introducing additional layers of organization. A well‐designed UI must facilitate navigation both across and within these hierarchical levels. It should also support playback and visualization of clips, while presenting relevant metadata for any selected item. These capabilities enable users to efficiently explore complex datasets and interpret CNN predictions in context.

#### 4.1.1 Design Pattern

The UI must provide effective tools for navigating both audio recordings and their associated clips. This is typically achieved through navigators, UI elements that allow analysts to select from a list of available options. While various types of navigators exist, a straightforward and intuitive approach involves a recording navigator, presented as an interactive table that lists available audio recordings. Once a recording is selected, a corresponding clip navigator can display the clips within that recording. This structure supports the integration of recognizer predictions. At the clip level, prediction scores can be shown directly within the clip navigator, either as numerical values or with color coding to support rapid visual interpretation. Since the recognizer processes audio at the clip level, raw predictions cannot be directly associated with the recording listings. However, a summary metric, such as the number of confirmed detections per recording, can be included in the recording navigator to provide quick insights.

For larger‐scale projects, the UI should support multiple recordings as input. In such cases, the recording navigator can be populated with the user‐defined sets of recordings, organized by project‐specific attributes such as location, date, or time of recording. This hierarchical structure enhances the efficiency of managing and analyzing complex acoustic datasets.

#### 4.1.2 Example

**FIGURE 4:**
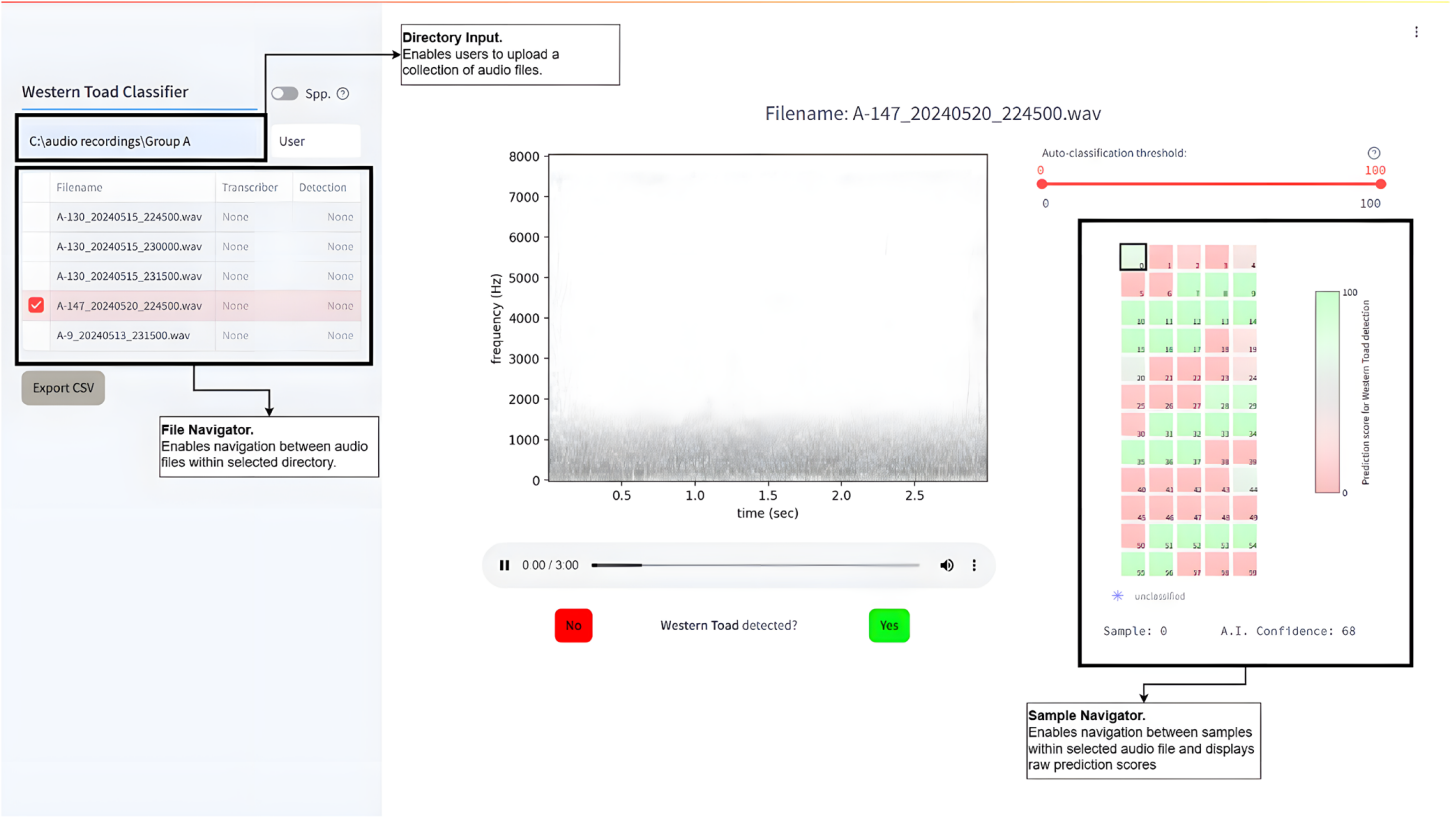
UI design elements that enable navigation between and within hierarchical bioacoustic datasets.

We developed a UI to facilitate and enhance analyst interaction with the western toad recognizer at multiple project scales (Figure 4). Analysts begin by specifying a directory containing audio recordings, which are then processed by the recognizer. The resulting predictions, along with the associated recordings, are used to populate the UI. This design allows analysts to define upper‐level project structures based on the organization of their folder hierarchy.

At finer project scales, the UI displays both a recording navigator and a clip navigator. The recording navigator is implemented as an interactive table, where each row represents a unique recording. Selecting a row loads the corresponding clips into the clip navigator, which is structured as an ordered grid of sequential clips. Selecting a clip triggers audio playback and displays its spectrogram representation.

In addition to navigation, the UI integrates recognizer predictions and manual verification across project scales. Within the clip navigator, predictions are visualized using a color gradient that fills the background of each grid cell, enabling quick identification of prediction distributions or high‐scoring clips. At the recording level, the recording navigator includes a dedicated column that flags the detection of a western toad in any of its clips. An export feature allows analysts to create a CSV summary of all detected western toad vocalizations across the dataset, supporting broader data aggregation and analysis.

### 4.2 Variable Effects of Threshold Values

When used alongside a threshold score, the predictions generated by a recognizer can support automatic classification. This approach can be used to identify clips that do not contain the species, those that do, or both (Stowell 2022). However, the choice of threshold value can significantly impact the outcome of any bioacoustic auto‐classification task (Knight and Bayne 2019). Low thresholds have been shown to produce more false positives, while high score thresholds increase the probability of missing the occurrence of a species (Knight and Bayne 2019). This trade‐off makes threshold selection a critical consideration in bioacoustic recognizer workflows.

The problem is further complicated in how recognizers assign prediction scores. The scores may not accurately reflect true probabilities, as recognizers can produce under‐ or over‐confident predictions. As a result, prediction scores may not be directly comparable across different species (Stowell 2022).

#### 4.2.1 Design Pattern

Given the trade‐off between type I and type II errors, as well as the variable relationship between prediction scores and true probability, any UI that supports automatic classification must allow analysts to manually adjust threshold values and inspect classifications across different thresholds. To enhance this functionality, the UI can support both a lower and an upper threshold. Under this dual‐threshold method, clips with prediction scores above the upper threshold are automatically classified as detections, while those below the lower threshold are considered absences. This approach helps mitigate the trade‐off between false positives and false negatives that typically arises when using a single threshold. However, clips with prediction scores falling between the two threshold values remain unclassified. For some applications, these unclassified clips may be negligible; in others, they can be flagged for manual review and classification.

#### 4.2.2 Example

**FIGURE 5:**
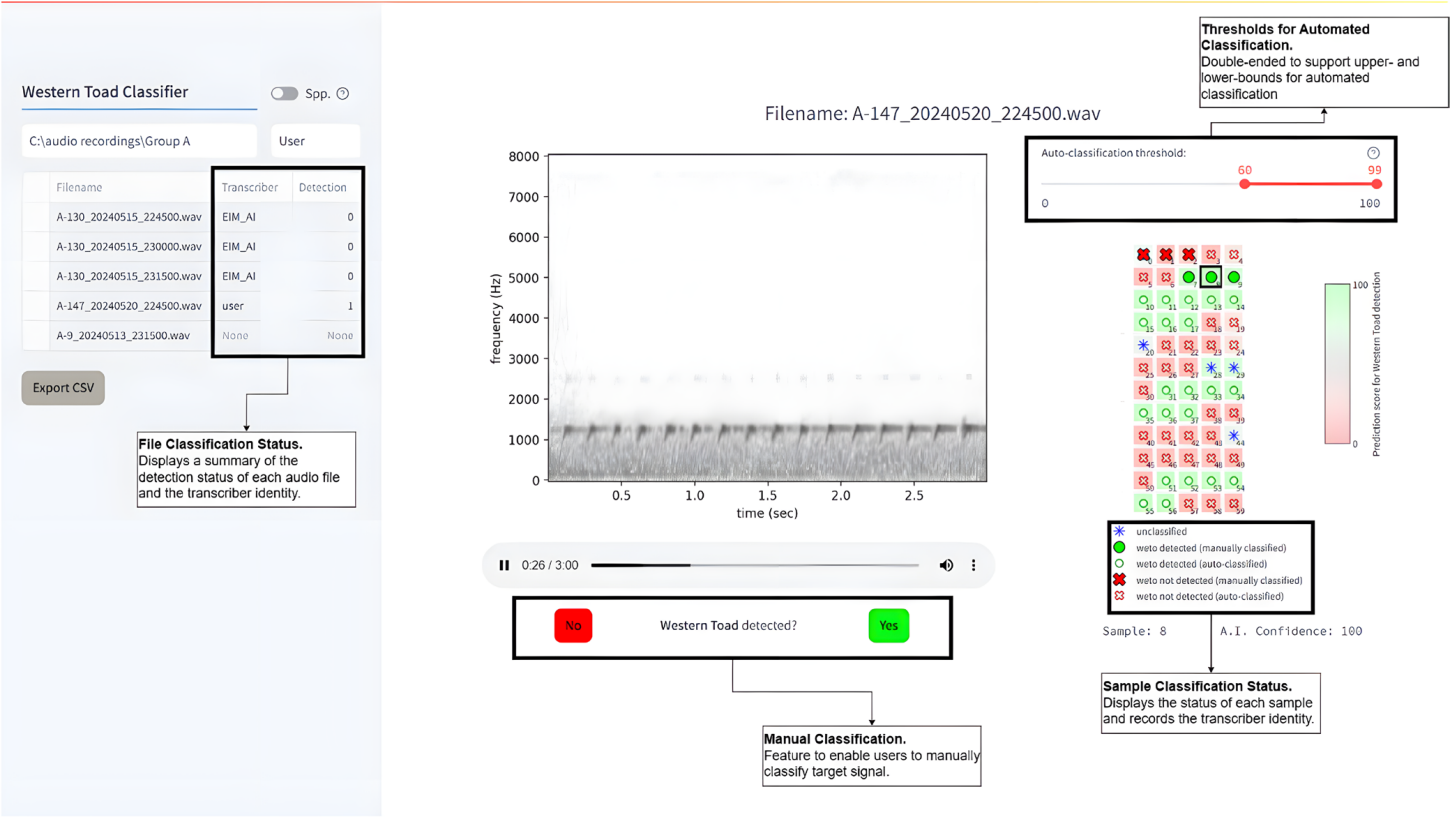
Features to support manual and automated classifications.

We implemented user‐defined lower and upper threshold for automated classification (Figure 5). Analysts interact with numerical inputs to set these bounds, which are then used to automatically classify all clips with prediction scores outside the specified range. Classification results are displayed in the clip navigator. At the recording level, a detection indicator is also displayed in one of the recording navigator's columns.

### 4.3 Supporting Manual and Automated Classifications

Many bioacoustic projects require the option to manually classify clips. Depending on the accuracy of recognizers, relying solely on them can compromise data quality. A well‐designed UI should support efficient manual review and provide a way to reconcile differences between manual and automated classifications.

#### 4.3.1 Design Pattern

Manual classification is most effective when analysts can review both acoustic and visual information together. Integrating spectrogram visualization with audio playback enables thorough inspection, while intuitive interface elements, such as buttons or dropdowns, reduce the likelihood of user error. Once a manual classification has been made, the UI needs to support its integration with automated classifications. The UI should record the source of each classification (e.g., recognizer or analyst). Manual classifications should take priority in cases of disagreement to reflect their higher reliability. At the recording level, summaries should follow clear rules: a species can be considered detected once any clip is marked as a detection but should not be marked absent until all clips have been reviewed without detection. Summaries or lists of detections can then be displayed for each recording.

#### 4.3.2 Example

In the Western Toad project, we implemented features to support manual classification (Figure 5). When a clip is selected, the corresponding audio playback and spectrogram are displayed, and analysts can record detections using color‐coded buttons. By default, clips are either tagged with a system‐generated transcriber label if they have been auto‐classified or left blank if unclassified. When a clip is manually classified, this label is replaced with the analyst's username, represented in the clip navigator with distinct symbols. In the recording navigator, transcriber information and detection status are summarized in dedicated fields. If a recording contains no detections but still has unclassified clips, both fields remain blank, signaling that review is incomplete. This design provides a transparent record of manual and automated classifications, supports efficient workflow, and helps maintain data quality.

### 4.4 Efficiency of Manual Classification

Manual classification tasks often require analysts to navigate large volumes of audio, with substantial time spent moving between recordings and individual clips. In bioacoustic projects, this can involve sifting through considerable noise for each detection. The repetitive nature of this task can reduce manual classification accuracy and increase analyst fatigue.

#### 4.4.1 Design Pattern

To improve efficiency, recognizer predictions can guide the order in which clips are presented for manual classification. Clips can be sorted through prediction score, so that clips most likely to contain detections are prioritized. Partially automating clip classification can further streamline workflow by including only unclassified clips for manual review. Optional automatic transitioning between recordings can also be implemented, but should remain under analyst control to avoid disrupting navigation within fully reviewed recordings. These principles help analysts to process large volumes of audio more efficiently.

#### 4.4.2 Example

**FIGURE 6:**
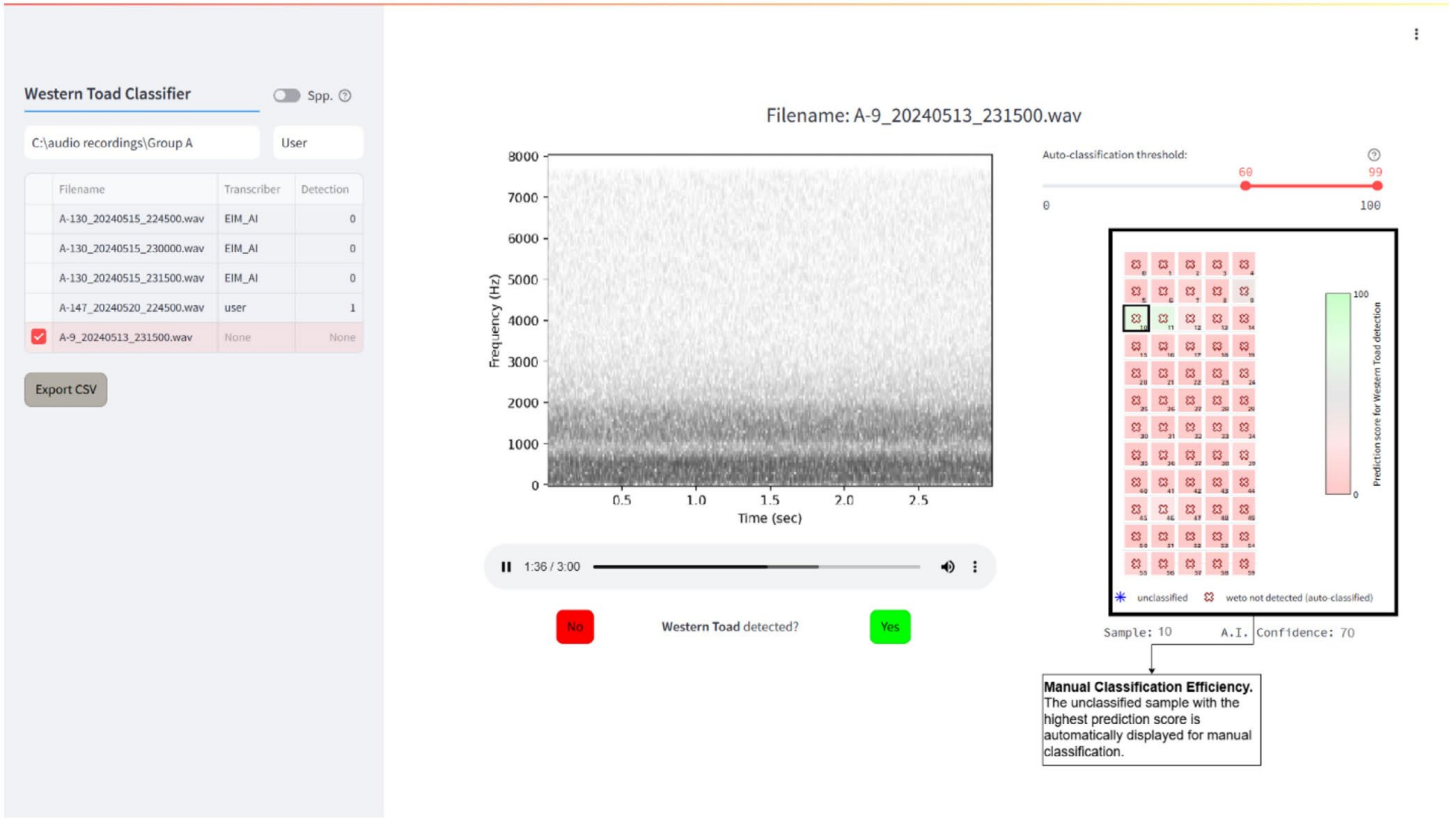
Automatic selection of unclassified clip with highest prediction score.

We designed the western toad bioacoustic UI to streamline analyst workflows (Figure 6). Clips are sorted by prediction score, and the highest‐ranking unclassified clip is presented first. Once a clip is manually classified, the next highest‐ranking unclassified clip is automatically displayed. We also included an optional feature that automatically advances to the next unclassified recording once the current recording is classified. This process continues until all recordings are classified or the analyst chooses to manually intervene.

### 4.5 Complexity of Multi‐Species Classification

As the number of species under consideration increases, classification tasks become more complex. This complexity arises because analysts must shift attention between species, rather than focusing on a single species at a time. As task complexity grows, so does the risk of human error, which can reduce the reliability of results.

#### 4.5.1 Design Pattern

To mitigate the challenges of multi‐species classification, workflows can be partitioned by species. A species selection feature allows analysts to focus on one species at a time, reducing the need to switch attention. Once a species is selected, the UI can reconfigure the recognizer predictions into a binary format, enabling a single‐species review process. Although classifying multiple species in parallel may be faster, a serialized approach simplifies the task, improving reliability. This design also accommodates analysts who are only qualified to classify a subset of species.

#### 4.5.2 Example

**FIGURE 7:**
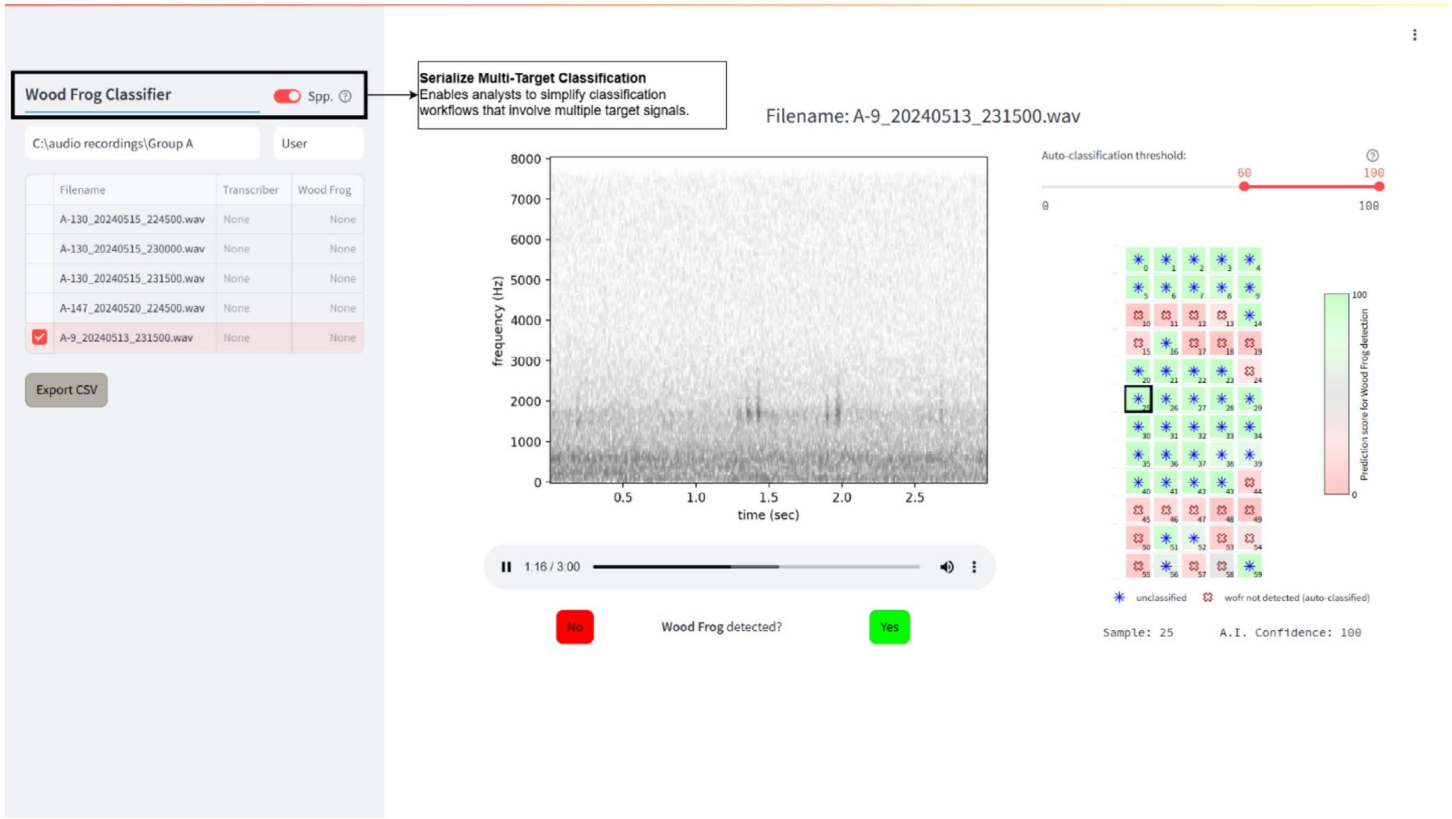
Serialize multi‐species classification tasks into binary workflows.

Although the UI developed for this project was focused on western toads, we additionally developed a recognizer for wood frogs in a related project. To manage both species in a single UI, we incorporated a toggle switch that allowed analysts to shift between single‐species binary workflows (Figure 7). Selecting a species adjusts the display and functionality accordingly, while all other design patterns remain unchanged. This approach maintains a consistent workflow and minimizes the potential for error when working with multiple species.

## 5 Discussion

Bioacoustic signal detection and classification projects benefit greatly from advances in machine learning. Automated recognizers can rapidly and accurately process large volumes of acoustic recordings, enabling expanded survey efforts at lower costs. While bioacoustics analysis for many species is supported through public software platforms or repositories, others require the development and integration of bespoke CNN recognizers. Developing state‐of‐the‐art CNN recognizers requires careful adherence to best practices in signal processing and machine learning. The design patterns discussed in Section 3 provide both conceptual and practical guidance across this process. Once developed, the full benefits of a CNN recognizer depend on its integration into a well‐designed UI. Although the optimal features of a bioacoustic UI depend on a study's goals, the patterns outlined in Section 4 highlight key principles for building effective UIs. Together, these patterns form a framework for an entire bioacoustic recognizer program, from initial design to implementation.

Importantly, the integration of recognizers into a UI creates opportunities for *active learning*, where model development and implementation become part of the same iterative process. Recognizers are first trained using available data, then deployed through the UI, where analysts review predictions, flag errors, and efficiently classify new data using predictions. The additional transcribed data can be used to retrain models. This process can be repeated, creating a continuous feedback loop that steadily improves performance. Through this process, the UI facilitates efficient classification and model refinement.

Automated recognizers can enhance a wide range of bioacoustic applications. They can be applied to any species that produce a distinct acoustic signal, including birds, cetaceans, terrestrial and aquatic mammals, anurans, insects, and fish (Brown 2024; Stowell 2022). Bioacoustic data have been used to measure biodiversity (Alcocer et al. 2022), estimate species densities (Marques et al. 2013), detect cryptic species (Lapp et al. 2021), and model occupancy (Czarnecki et al. 2024), distribution (Ribeiro et al. 2022), and migratory movements (Van Doren et al. 2024). In BNP, bioacoustic surveys are used to model Western Toad occupancy. The integration of an automated recognizer has enabled a substantial expansion of survey effort and improved the precision of occupancy models. Beyond enhancing ongoing bioacoustic programs, the reduced cost of automated surveys makes large‐scale efforts feasible even in resource‐limited settings.

Machine learning offers opportunities beyond bioacoustics. Computer vision tools have been developed to automate camera trap image classification and quantify vegetation abundance and composition in small‐scale quadrats (Beery et al. 2019; McCool et al. 2018). As these tools evolve, they are likely to drive further innovation across ecological research. Although this paper focuses on bioacoustics, many of the design patterns discussed here can be adapted to other ecological disciplines. Thoughtful development and implementation of machine learning tools hold strong potential for enhancing ecological programs and improving our understanding and management of ecosystems.

## Author Contributions

**Gavin Hurd:** data curation (supporting), formal analysis (lead), writing – original draft (lead), writing – review and editing (equal). **Robin Baron:** conceptualization (equal), data curation (lead), writing – review and editing (equal). **Jesse Whittington:** conceptualization (equal), supervision (lead), writing – review and editing (equal).

## Funding

The authors have nothing to report.

## Conflicts of Interest

The authors declare no conflicts of interest.

## Data Availability

All data and code supporting the designs and workflows described in this study are publicly accessible. The convolutional neural network recognizer training scripts are archived on Zenodo and are available at https://doi.org/10.5281/zenodo.17993137. The source code for the user interface is archived on Zenodo and is available at https://doi.org/10.5281/zenodo.17993133. A subset of the raw audio recordings used in this study is archived on Zenodo to facilitate replication and review and is available at https://doi.org/10.5281/zenodo.17993187.
